# Clinicopathological features and management of biliary cystic tumors of the liver: a single-center experience

**DOI:** 10.1007/s00423-023-02994-2

**Published:** 2023-07-11

**Authors:** El-Sayed Abou El-Magd, Mohamed El-Shobari, Ramy A. Abdelsalam, Amr Abbas, Youssif Elmahdy, Hosam Hamed

**Affiliations:** 1 Department of General Surgery, Faculty of Medicine, Gastrointestinal Surgical Center GISC, Mansoura University, Gehan Street, Mansoura, Al Dakahlia Governorate 35511 Egypt; 2 Pathology Department, Faculty of Medicine, Mansoura University, Mansoura, Egypt

**Keywords:** Mucinous biliary cystic tumors, Biliary cystadenoma, Biliary cystadenocarcinoma, Clinicopathological criteria, Surgical outcomes

## Abstract

**Background:**

Biliary cystic neoplasms (BCNs) of the liver are rare pathologies encountered in hepatobiliary surgeries. Till now, there is a lack of definitive criteria used to differentiate biliary cystadenoma (BCA) from biliary cystadenocarcinoma (BCAC).

**Methods:**

In the period between 2005 and 2018, the data of consecutive patients diagnosed with BCA and BCAC were retrospectively reviewed.

**Results:**

A total of 62 patients underwent surgical management for BCNs. BCA was diagnosed in 50 patients while 12 patients had BCAC. Old age, male gender, smoking, and abdominal pain were strongly associated with BCAC. Left lobe location, small size, with the presence of mural nodule, and solid component were significantly noticed with BCAC. A novel pre-operative score was developed to predict the susceptibility for BCAC and help us to identify the optimal surgical strategy. Blood loss, operative time, and complications were comparable between the two study groups.

**Conclusion:**

Mural nodules or solid components are suggestive of BCAC. Complete surgical resection of cystic tumors of the liver is mandatory due to malignant potential of the lesion and for prolonged survival.

## Introduction

Intrahepatic biliary cystic neoplasms of the liver are rare in clinical practice, as it accounts for 5–10% of intrahepatic cystic lesions of biliary origin [1–4] and about 1% of all hepatic cystic lesions [5]. These tumors include biliary cystadenoma and biliary cystadenocarcinoma [6]. Some surgeons believe that the previous incidence is underestimated, as some of these lesions are often misdiagnosed as simple liver cysts [7].

These tumors arise from an aberrant bile duct or from a primitive hepatobiliary stem cell [8]. The diagnosis of these cystic lesions generally depends on radiological imaging criteria, including thick-walled cyst, with multiloculs, septations, mural nodules, and irregular or smooth walls [6, 9].

In spite of the tremendous advances in the field of radiological imaging, it remains inconclusive to distinguish between BCA and BCAC using conventional imaging techniques like ultrasonography (USG), computed tomography (CT), or magnetic resonance imaging (MRI) [2, 10, 11].

Owing to the rare nature of these cysts, there is an apparent lack of reliable clinical and radiological criteria differentiating BCA from BCAC [8]. Reaching affirmative data regarding the previous perspectives will help the surgeon to choose the optimal surgical resection technique.

We aimed to assess the clinical and radiological characteristics of BCA and BCAC and analyze their post-resection outcomes. In addition, we wanted to define the disease-free survival (DFS) and overall survival (OS) of patients with BCNs relative to the type of surgical procedure performed (e.g., unroofing/fenestration vs hepatic resection), as well as the pathological data.

## Patients and methods

This is a retrospective cohort study of all patients who underwent surgical management for hepatic BCNs between January 2005 and December 2018 at Gastrointestinal Surgery Center, Mansoura University, Egypt. After pathological examinations by experienced pathologists, biliary cystic neoplasms were classified into BCA and BCAC. Exclusion criteria included patients who had inconclusive pathological examination or were not managed by surgical resection. The study was approved by the institutional review board of Faculty of Medicine, Mansoura University (IRB code: R.22.04.1694).

Patients with hepatic cystic lesions were diagnosed by abdominal ultrasound (US) and triphasic abdominal computerized tomography (CT). If the diagnosis could not be established, further evaluation by MRI was requested. Cardinal features for radiological diagnosis were the presence of a multilobular cystic lesion, with well-defined capsule and the presence of one or more of the following structures features: internal septation with nodular areas, wall thickness irregularities, mural nodules, and calcification along the wall. Diagnostic liver biopsy or percutaneous cyst drainage was not adopted routinely in this series. All patients signed informed consent before the surgical procedure.

Surgical management was performed through either open or laparoscopic approach. Management strategies included deroofing, enucleation, or radical resection with safety margin based on the type of the cystic tumor based on radiological evaluation.

Regular follow-up visits were scheduled annually after surgery. Follow up was performed through clinical assessment and abdominal ultrasound. If recurrence was suspected on abdominal US, further evaluation by abdominal CT and MRI was performed. Post-operative mortality was also recorded.

Patients’ data were retrieved from a prospectively maintained database including demographic data, clinical presentation, liver functions tests, tumor markers, and findings on imaging studies. Operative data included management technique, operative time, blood loss, and operative difficulties. Postoperative data included surgical complications as biliary leakage, surgical site infection, collections, and bleeding.

### Statistical analysis

The collected data was revised, coded, tabulated, and introduced to a PC using Statistical package for Social Science (IBM Corp. Released 2017. IBM SPSS Statistics for Windows, Version 25.0. Armonk, NY: IBM Corp.). Student *t* test was used to assess the statistical significance of the difference between two study group means. The Kruskal-Wallis test was used to assess the statistical significance of the difference between more than two study group non-parametric variables. Chi-square test was used to examine the relationship between two qualitative variables. Kaplan–Meier test was used for survival analysis, and the statistical significance of differences among curves was determined by log-rank test. Cox regression analysis was used for prediction of risk factors affecting survival. Logistic regression analysis was used for prediction of risk factors, using generalized linear models. The ROC curve (receiver operating characteristic) provides a useful way to evaluate the sensitivity and specificity for quantitative diagnostic measures that categorize cases into one of two groups. The optimum cut off point was defined as that which maximized the area under ROC curve (AUC) value. All reported *p* values were two-tailed and *p* < 0.05 was considered to be significant.

## Results

### Demographic and clinical data

Between January 2005 and December 2018, a total of 62 patients underwent surgical management for hepatic BCNs. After pathological examinations, BCA was diagnosed in 50 patients while 12 patients had BCAC.

The mean age of the study population was 44.2 ± 13.3 years, and the male to female ratio was 1:1.7. Demographic, clinical, and laboratory data of the study population is summarized in Table 1. Smoking and older age were significantly associated with BCAC compared to BCA group (*p* < 0.001). Abdominal pain was significantly higher in the BCAC group (*n* = 12/12) compared to the BCA group (*n* = 31/50) (*P* = 0.012).

**Table 1:** Demographic and baseline characteristics

		All patients (*n*= 62)	Cystadenoma (*n*= 50)	Cystadenocarcinoma (*n*= 12)	*P*
Age (years)		44.2 ± 13.3	40 ± 12.3	61.8 ± 10.1	**< 0.001**
Gender	Male	23(37.1%)	14(28%)	9(75%)	**0.006**
	Female	39(62.9%)	36(72%)	3(25%)	
History of smoking		9(14.5%)	0(0%)	9(75%)	**< 0.001**
Duration of symptoms		4(1−24)	10(2−24)	2(1−2)	0.299
Symptoms	Asymptomatic	19(35.5%)	19(38%)	0(0%)	0.512
	Symptomatic	43(69.4%)	31(62%)	12(100%)	
Abdominal pain		43(69.4%)	31(62%)	12(100%)	**0.012**
CEA (ng/mL)		2(0.5−5)	2(0.5−5)	1.7(1.4−5)	0.902
CA19_9 (U/mL)		10(2−895)	10(2−895)	25.5(2.5−200)	0.099
AFP (μg/L)		2(1−7)	2(1−7)	2.4(2−6.3)	0.187
SGOT		21(20−106)	21(20−106)	20(20−25)	0.505
SGPT		23(20−160)	22(20−160)	24(20−31)	0.925
T Bil (mg/dL)		0.5(0.4−3.9)	0.5(0.4−0.8)	0.4(0.4−3.9)	0.762
ALP		5(5−13)	5(5−13)	6(5−7)	0.148

Most of the collected laboratory data, including liver function tests and tumor markers, showed no significant difference between the two groups (*p* > 0.05) (Table 1).

There was no statistically significant difference in the pre-operative blood levels of carbohydrate antigen 19-9 (CA19-9; *p* = 0.09), carcinoembryonic antigen (CEA; *p* = 0.9), or α-fetoprotein (AFP; *p* = 0.18) between the BCA and BCAC groups. Seven patients in the BCA group and two in the BCAC group had elevated CA19-9.

Only one patient in the BCAC group and no one in the BCA group had elevated total bilirubin. There were no statistically significant differences in the levels of total bilirubin (*p* = 0.76), SGPT (*p* = 0.92), and alkaline phosphatase (ALP; *p* = 0.14) between the two groups.

### Pre-operative radiological diagnosis

Eight patients were referred after undergoing percutaneous liver biopsy and aspiration cytology, and this leads to inconclusive diagnosis. Abdominal MRI was requested in 20 patients for further differentiation between BCAC and BCA. Regarding tumor location, thirty five (56.5%) cases were located in the left lobe (BCA: 24 (48%), BCAC: 11 (91.7%)); twenty six cases (41.9%) were in the right lobe (BCA: 25(50%), BAC: 1 (8.3%)), while one case of BCA (1.6%)) was located in the caudate lobe.

**Fig. 1:**
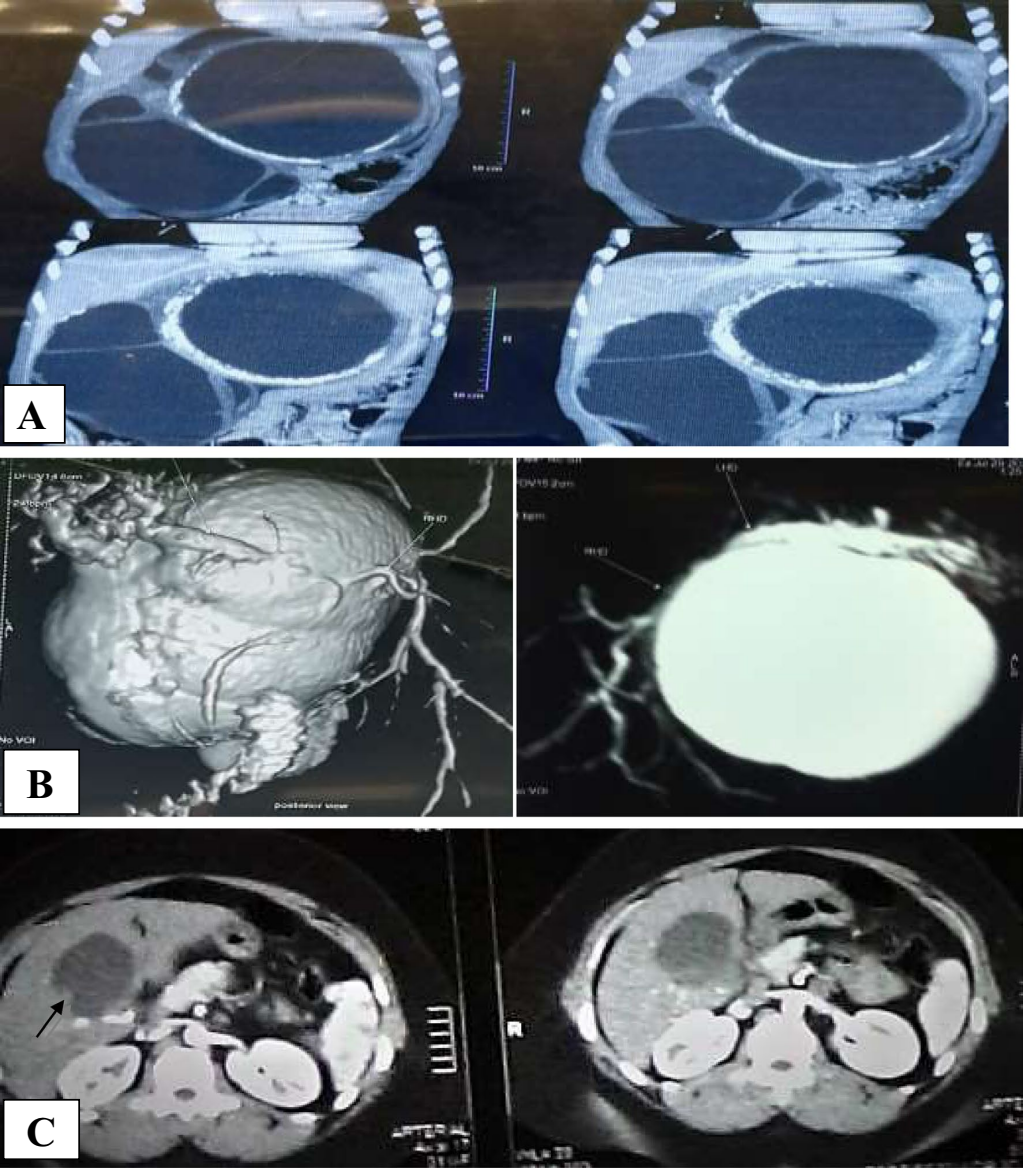
**A** Biliary cystic tumor associated with multiple loculation and mural calcifications. **B** Biliary cystic tumor associated with IHB dilatation. **C** Biliary cystic tumor associated with mural nodule

Findings on various imaging modalities are summarized in Table 2. Findings on imaging studies (Fig. 1), which were statistically associated with a diagnosis of BCAC, were smaller tumor size (*p* = 0.001), left-sided (*p* = 0.012), the presence of mural nodules (*p* = 0.03), mural calcifications (*p* = 0.02), solid component (*p* = 0.04), and the presence of intrahepatic biliary dilatation (*p* = 0.013). Nevertheless, the cyst septation, loculation, hypervascularity, enhancement, and wall thickness did not show a significant difference between the two groups.

**Table 2:** Finding on imaging studies

		All patients (*n*= 62)		Cystadenoma (*n*= 50)	Cystadenocarcinoma (*n*= 12)	*P*
Radiology	CT & US	42(67.7%)		31(62%)	11(91.7%)	**0.083**
	CT, US & MRI	20(32.3%)		19(38%)	1(8.3%)	
Cyst size (cm)		8(1−20)		10(1−20)	5(5−12)	**< 0.001**
Septation		53(85.5%)		42(84%)	11(91.7%)	0.675
Loculation		51(82.3%)		41(82%)	10(83.3%)	0.914
Hypervascularity		25(40.3%)		18(36%)	7(58.3%)	0.198
Wall thickness		Thin	27(43.5%)	22(44%)	5(41.7%)	0.884
		Thick	35(56.5%)	28(56%)	7(58.3%)	
Mural nodule		3(4.8%)		1(2%)	2(16.7%)	**0.033**
Solid component		1(1.6%)		0(0%)	1(8.3%)	**0.040**
Mural calcification		10(16.1%)		5(10%)	5(41.7%)	**0.018**
Associated IHBD		13(21%)		7(14%)	6(50%)	**0.013**
Cyst location		Left lobe	35(56.5%)	24(48%)	11(91.7%)	**0.012**
		Right lobe	26(41.9%)	25(50%)	1(8.3%)	
		Caudate lobe	1(1.6%)	1(2%)	0(0%)	
Enhanced wall		No	28(45.2%)	23(46.0%)	5(41.7%)	0.786
		yes	34(54.8%)	27(54.0%)	7(58.3%)

Based on radiological evaluation, thirteen patients were misdiagnosed as simple liver cyst instead of BCA and underwent inappropriate treatments including percutaneous transcatheter drainage (*n* = 7) and laparoscopic fenestration (*n* = 6). In the BCAC group, two patients were misdiagnosed preoperatively as BCA.

### Pre-operative score for differentiation between BCA and BCAC

Regression analysis was conducted for prediction of cystadenocarcinoma susceptibility, using age, gender, smoking, abdominal pain, duration, CEA, CA19-9, left sided, vascularity, and IHBD as covariates as demonstrated in Table 3.

**Table 3:** Regression analysis for prediction of cystadenocarcinoma susceptibility

	Univariable				Multivariable			
	*p*	OR	95% CI		*p*	OR	95% CI	
Age	.001	1.066	1.026	1.107	.024	1.004	1.001	1.008
Male versus female	.004	3.858	1.541	9.658	.023	1.259	1.120	1.319
Smoking	.044	1.653	1.118	2.976	.003	1.246	1.076	1.442
Abdominal pain	.555	.653	.159	2.687				
Duration	.023	.102	.014	.726	.005	.991	.985	.997
CEA	.017	1.161	1.039	1.823	.048	1.379	1.148	1.858
CA19-9	.042	1.302	1.196	1.508	.049	1.202	1.196	1.709
Left sided versus non left sided	.005	4.923	1.633	14.841	.026	1.426	1.102	1.768
Vascularity	.556	1.288	.554	2.995				
Size	.014	.754	.601	.945	< 0.001	.978	.968	.989
Associated IHBD	.016	3.395	1.256	9.179	.014	1.144	1.097	1.313
Mural nodules	0.376	1.287	0.859	1.857				
Mural calcification	0.467	1.870	0.698	2.387				
Solid component	0.609	1.298	0.967	1.376				

Older age, male gender, smokers, and shorter duration and higher CEA, CA19-9, left sided, smaller tumor size, and associated IHBD dilatation were associated with risk of cystadenocarcinoma susceptibility in uni- as well as multivariate analyses.

Regression analysis was conducted to create a score for discrimination between BCA and BCAC. The incorporating all factors provided the best overall accuracy: (Table 4).

**Table 4:** Score for differentiation between biliary cystadenoma and cystadenocarcinoma

Risk factors		Score coefficients
Intercept		0.158
Age		0.004
Gender	Female = 0 Male = 1	0.058
Smoking		0.220
Duration		-0.008
CEA		0.149
CA19-9		0.002
Location	Left = 1 Non left = 0	1.594
Size		-0.027
Associated IHBD dilatation	Absent = 0 Present = 1	1.222

$$Score=0.158+\left[\left( age\times 0.004\right)+\left( male\times 0.058\right)+\left( smoking\times 0.220\right)\right)+\left( CEA\times 0.149\right)+\left( CA19-9\times 0.002\right)+\left( left\ sided\times 1.594\right)+\left( dilated\ IHBD\times 1.222\right)-\left(\left( duration\times 0.008\right)+\left( size\times 0.027\right)\right)\Big].$$

**Fig. 2:**
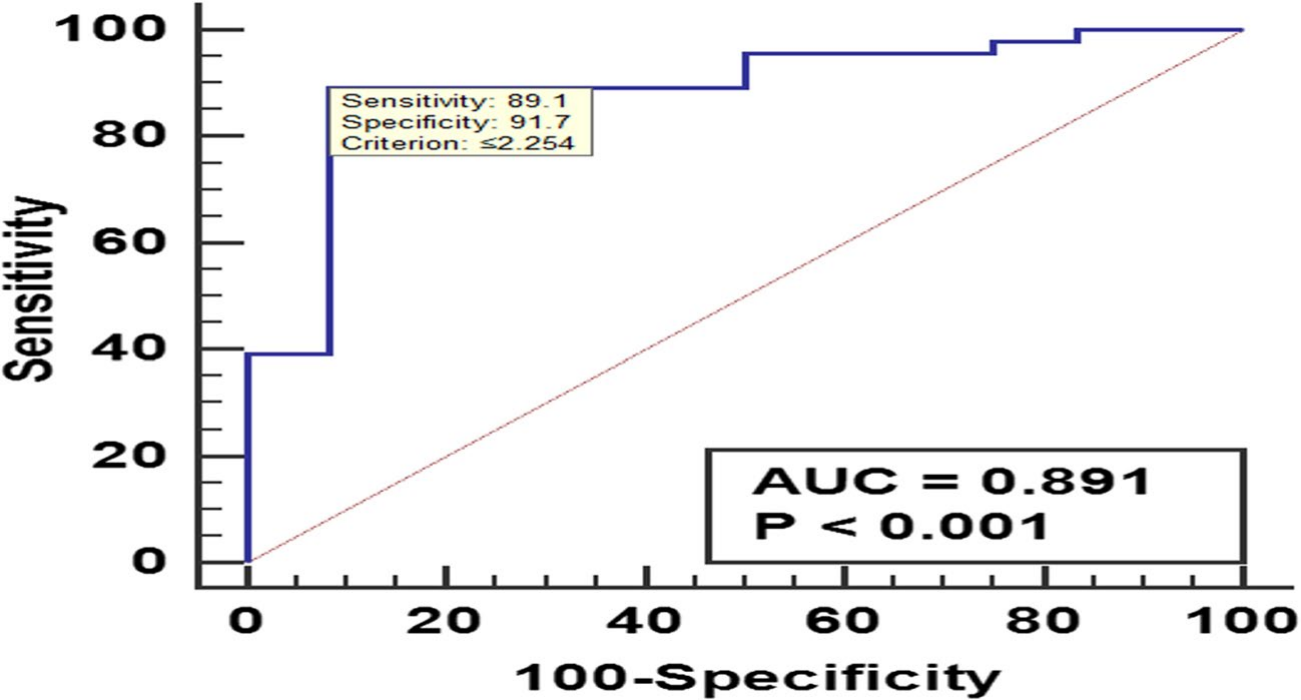
ROC curve of the score for discrimination between biliary cystadenoma and cystadenocarcinoma

A ROC curve of the created score was conducted to provide the best cut off value as well as the performance characteristics of the score for discrimination between BCA and BCAC. Score best cut off value was 2.25 for prediction of BCAC among studied patients, with 89.1% sensitivity, 100% specificity (Fig. 2).

### Surgical management and post-operative outcome

**Fig. 3:**
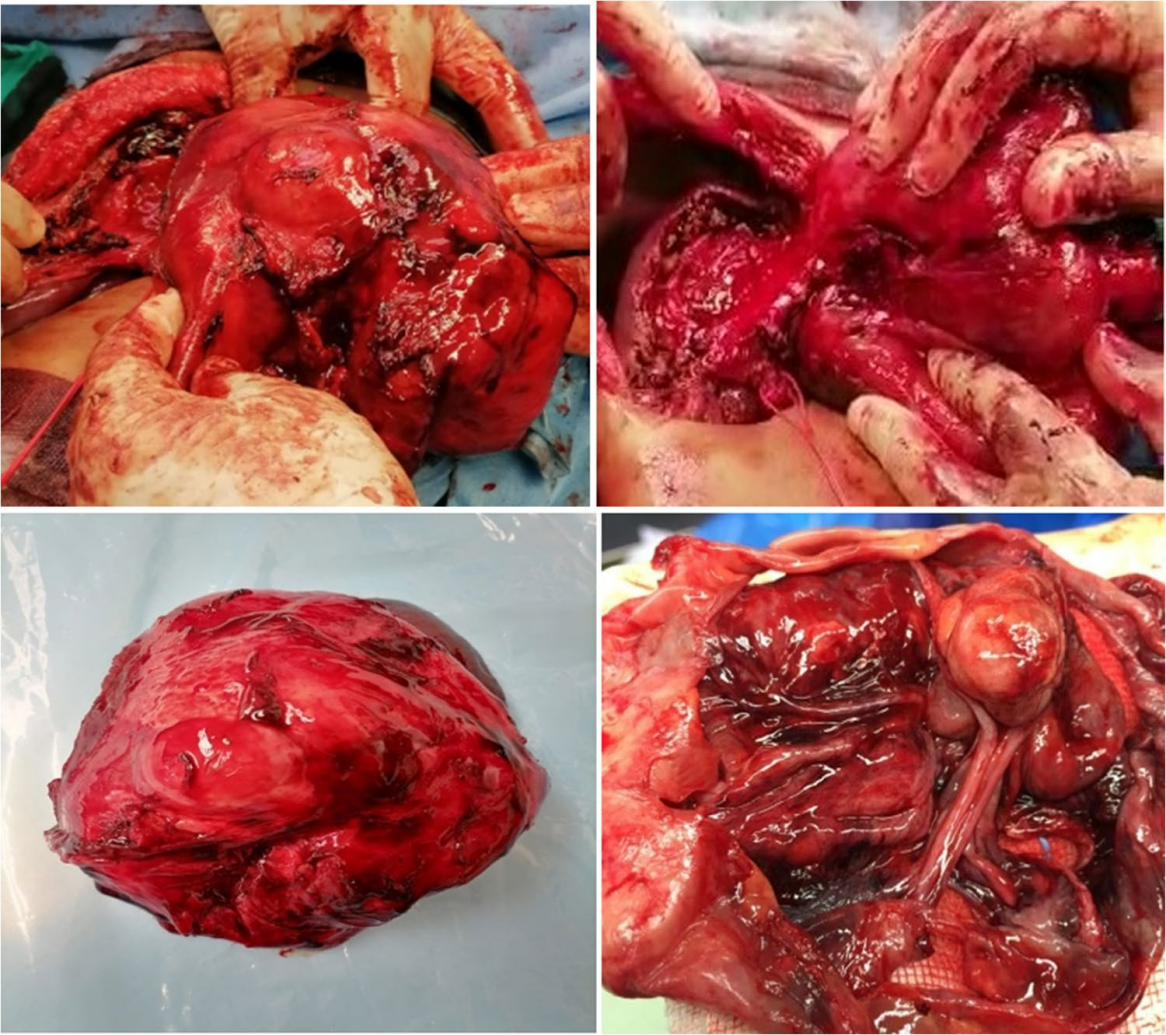
Left lobe BCAC with mural nodules managed by left hepatectomy

**Fig. 4:**
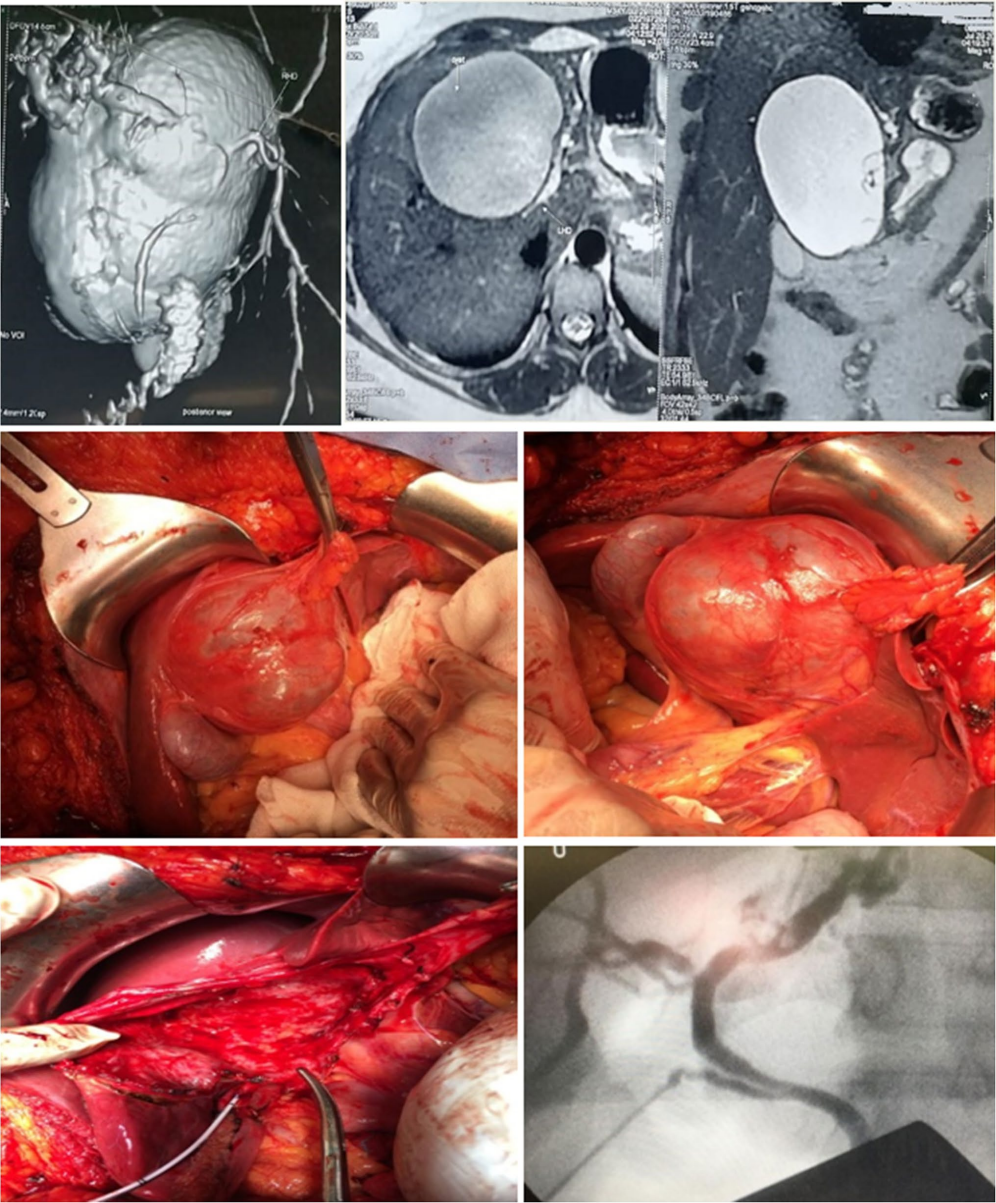
Radiological and operative view of Segment IV BCA associated with biliary compression

**Fig. 5:**
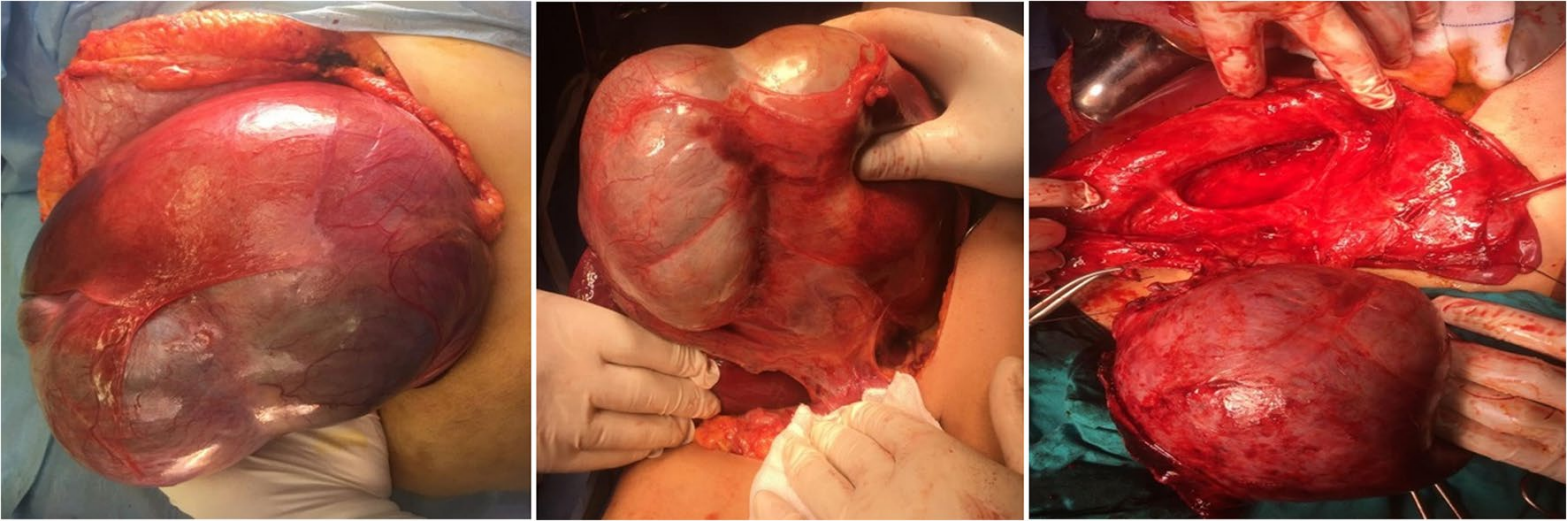
Operative view of huge lt lobe BCA underwent enucleation

**Fig. 6:**
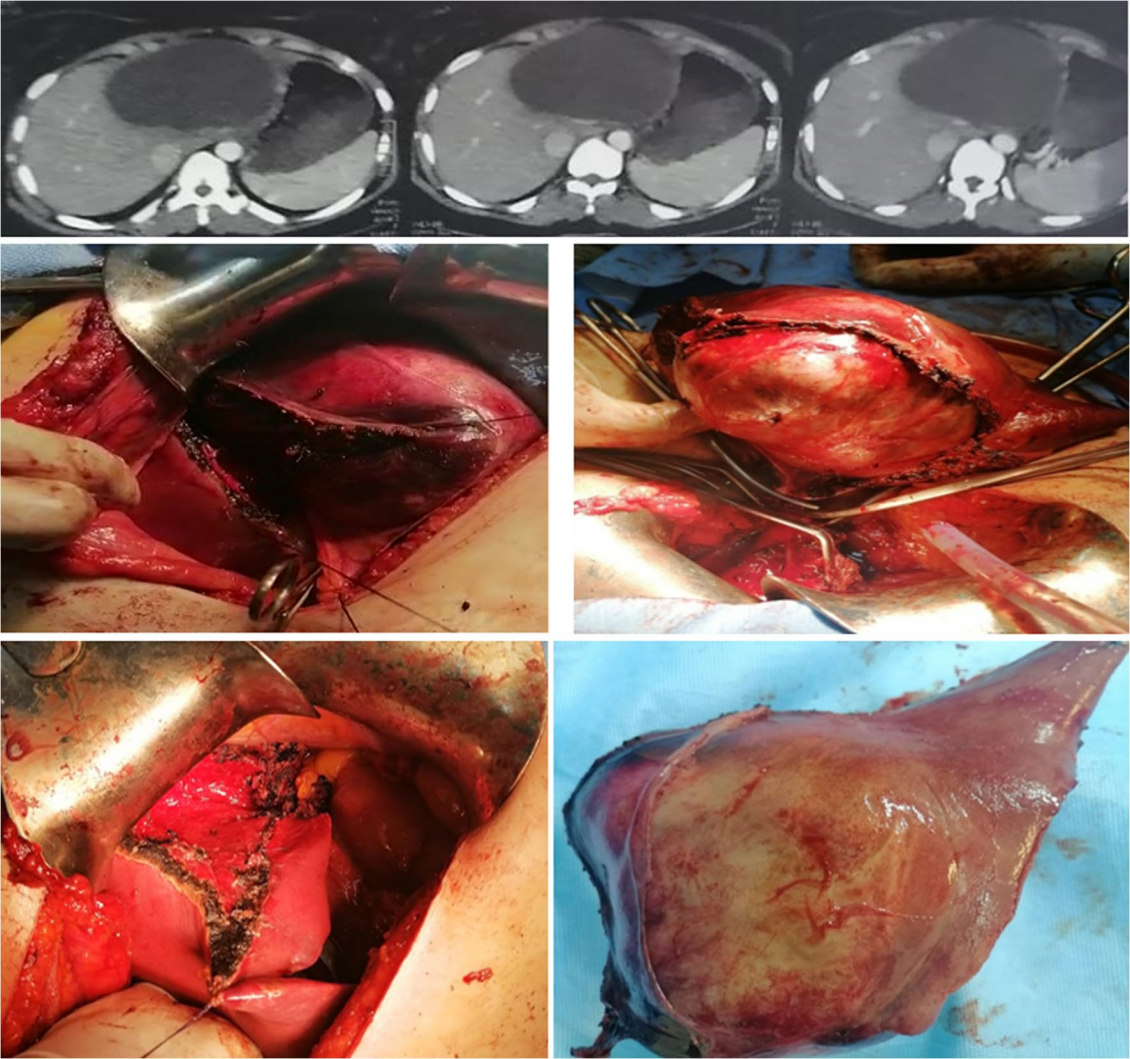
Radiological and operative view of left lobe BCA underwent left hepatectomy

For BCAC group surgeries performed including enucleation (*n* = 4) and radical resection including left lateral sectionectomy (*n* = 4), left hemihepatectomy (*n* = 3), (Fig. 3), and right hemihepatectomy (*n* = 1). For BCA, group surgeries were successfully performed including fenestration and deroofing (*n* = 6 patients misdiagnosed as simple liver cyst), enucleation (*n* = 38) (Figs. 4 and 5), and major resections including left hemihepatectomy (*n* = 1) (Fig. 8), left lateral sectionectomy (*n* = 2), right hemihepatectomy (*n* = 1), and right posterior sectionectomy (*n* = 2) (Fig. 6).

Laparoscopic approach was adopted in 18 patients (29%) in the BCA group for laparoscopic fenestration and deroofing (*n* = 4) and laparoscopic enucleation (*n* = 14). Most malignant patients were managed by radical resection with safety margin (66.7%), while most benign ones were managed by enucleation. That is why the operative decision was significantly different between the two groups (*p* = 0.001).

Operative time and blood loss were statistically comparable between the two groups. Operative data and postoperative outcome measures are summarized in Table 5.

**Table 5:** Operative details and post-operative outcome

		All patients (*n*= 62)	Cystadenoma (*n*= 50)	Cystadenocarcinoma (*n*= 12)	*P*
Operation	Enucleation	42(67.7%)	38(76%)	4(33.3%)	**< 0.001**
	Radical resection	14(22.6%)	6(12%)	8(66.7%)	
	Fenestration & deroofing	6(9.7%)	6(12%)	0(0%)	
Approach	Open	44(71%)	32(64%)	12(100%)	**0.013**
	Laparoscopic	18(29%)	18(36%)	0(0%)	
Blood loss (ml)		250(50−2000)	250(50−2000)	350(200−1500)	0.159
Operative time (minutes)		150(90−300)	180(90−300)	150(120−280)	0.730
Complications	Bleeding	0(0%)	0(0%)	0(0%)	-
	Collection	1(1.6%)	1(2%)	0(0%)	0.621
	Bile leak	4(6.5%)	3(6%)	1(8.3%)	0.768
Hospital stay (days)		5(3−13)	5(3−10)	5(4−13)	0.225
Follow-up period (months)			64.2 ± 22	47.4 ± 8.8	**0.025**
Recurrence		17(27.4%)	6(12%)	11(91.7%)	**< 0.001**

Only in the BCA group, there was intra-operative bile duct injury in 6 patients, managed by primary repair (right main duct in 2 patients, left main duct in 3 patients, and common hepatic duct in one patient). The incidence of post-operative complications and the duration of hospitalization were statistically comparable between the two groups.

Post-operative bile leakage developed in 4 patients, two of them managed by ERCP and stent insertion and the other two resolved with conservative management. Table 4 shows the previous data.

### Pathological evaluations

Pathological diagnosis of BCA was confirmed by the following characteristics, multilobulated cysts lined by mucinous epithelial cells overlying ovarian type stroma. While diagnosis of BCAC was confirmed by mucinous epithelial cells exhibit malignant criteria (frequent mitotic figures, nuclear pleomorphism, complex tubulopapillary projections) with or without ovarian type stroma.

**Fig. 7:**
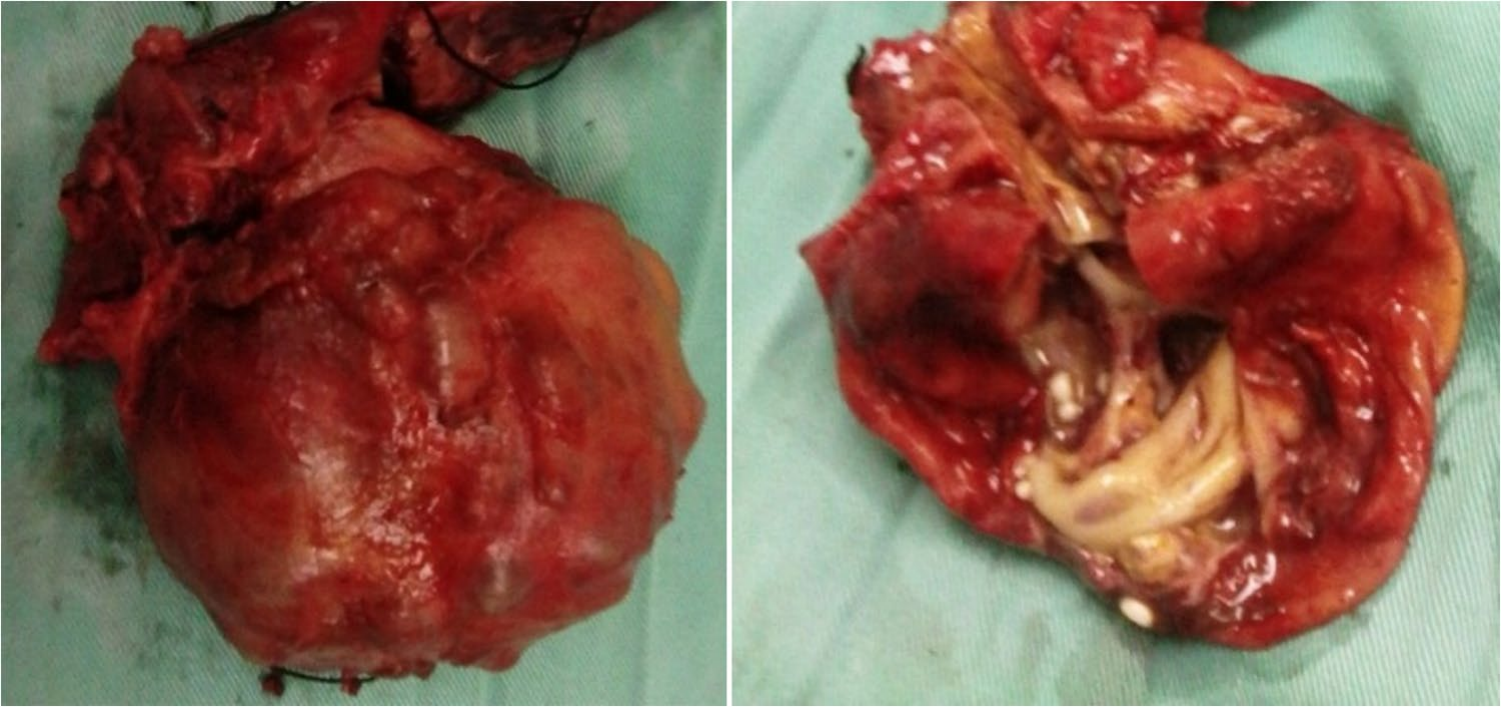
BCAC with infiltrative solid component to the nearby liver tissue

After final pathological assessment, most of the mucinous BCNs lesions were noted to be BCA 50 (80.7%), whereas 12 (19.3%) were BCAC. In the BCAC group, the lesion has the following features, multilobulated cysts lined by mucinous epithelial cells and ovarian type stroma in 41.7% of patients, but associated with liver tissue invasion in 75% of patients (Fig. 7; Table 6).

**Table 6:** Pathological evaluation of the studied groups

	All patients (*n* = 62)		Cystadenoma (*n* = 50)	Cystadenocarcinoma (*n* = 12)	*P*
Ovarian_stroma	No	7(11.3%)	0(0.0%)	7(58.3%)	0.001
	Yes	55(88.5%)	50(100%)	5(41.7%)	
Tissue_invasion	No	3(25.0%)	0(0.0%)	3(25.0%)	–
	Yes	9(75.0%)	0(0.0%)	9(75.0%)	

### Follow up recurrence and survival

**Fig. 8:**
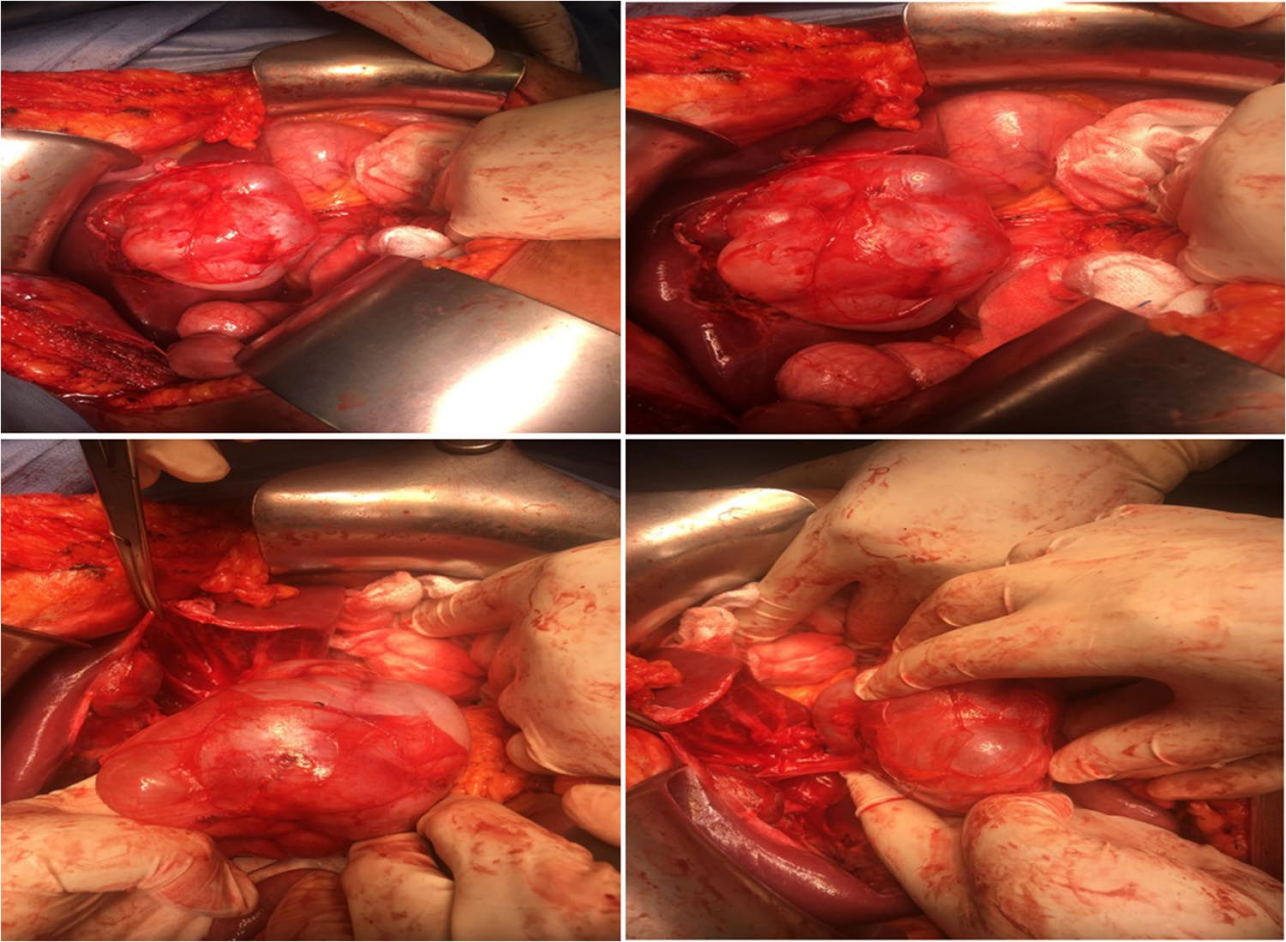
Operative view of recurrent Seg IV BCA after previous fenestration, managed by enucleation

For the BCA group, the mean follow-up period was (64.2 ± 22 months). There was recurrence of the disease in 6 (12%) patients previously managed by deroofing or fenestration. In the BCA group, an increased likelihood of recurrence was associated with the type of surgical procedure performed. Specifically, the incidence of recurrence was 100% among patients who underwent an unroofing/fenestration (Fig. 8). All of those 6 patients, who recurred, underwent repeat resection. Repeat surgery involved partial hepatic resection in four patients (66.7%) and hemi hepatic resection in the other two patients (33.3%).

**Fig. 9:**
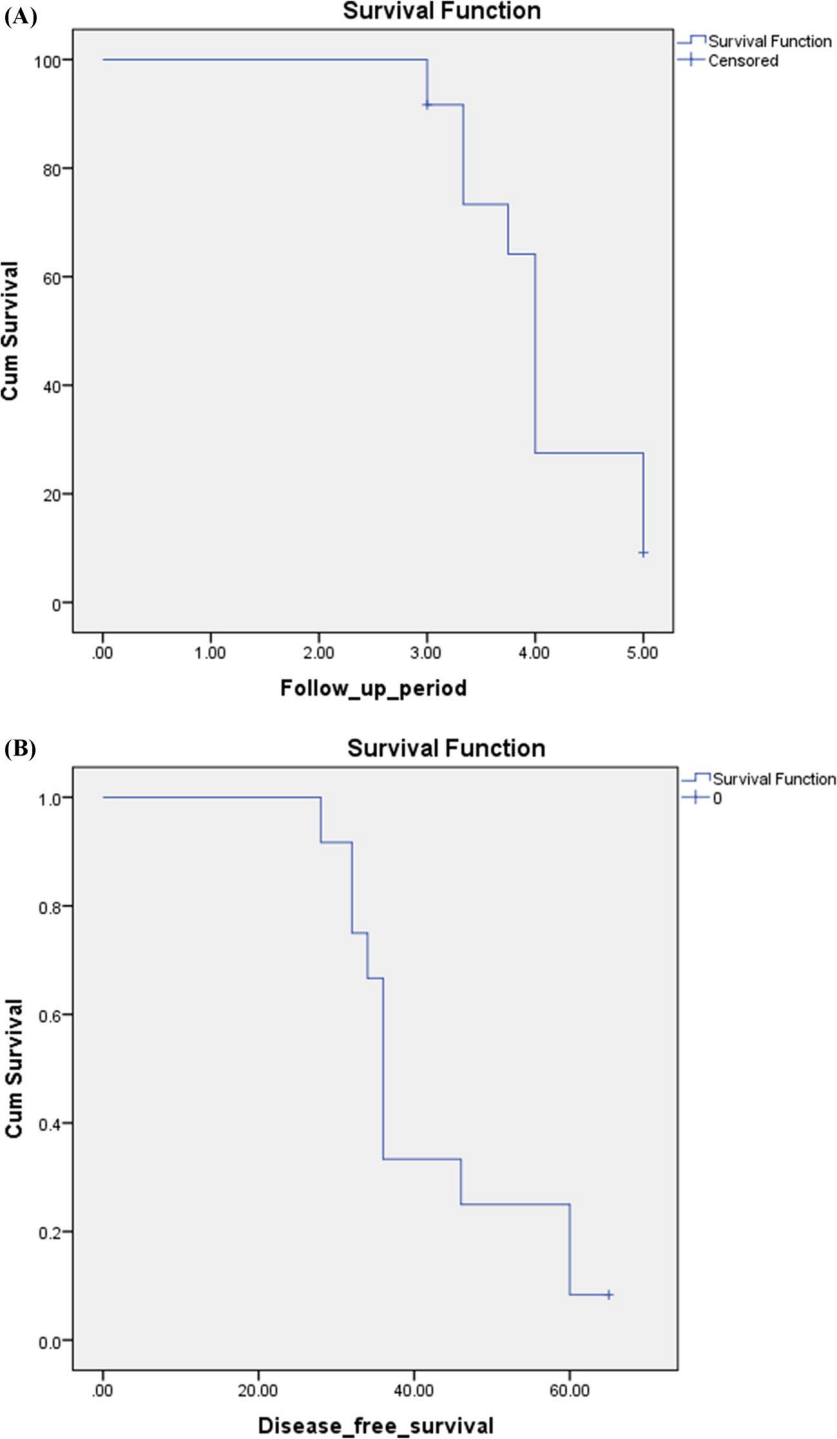
Overall (**A**) and **B** disease-free patient survival curves of patients with biliary cystadenocarcinoma

For the BCAC group, the mean follow-up period was 47.4 ± 8.8 months. The overall incidence of recurrence was generally higher among patients with BCAC (91.7 %) versus BCA (12%) (*P* < 0.001), regardless of the procedure performed. Disease-free survival rate (DFS) was 100%, 33.3%, and 8.3%, at 1 year, 3 years, and 5 years respectively, whereas overall survival rate was 100%, 91.7%, and 9.2%, at 1 year, 3 years, and 5 years respectively (Table 7; Fig. 9).

**Table 7:** Disease survival in the cystadenocarcinoma group

	Cystadenocarcinoma patients (*n* = 12)	
	OS	DFS
Median survival	48 months	36 months
One-year survival	100%	100%
Three-year survival	91.7 %	33.3%
Five-year survival	9.2%	8.3%

Cox regression analysis was conducted for prediction of DFS, using age, gender, operation, tissue invasion, ovarian stroma, and pathology data as confounders. Older age, tissue invasion, and absent ovarian like stroma and lymphatic infiltration were associated with shorter DFS in univariable analysis. However, in multivariable analysis, only tissue invasion and lymphatic infiltration were considered independent predictors of shorter DFS (Table 8).

**Table 8:** Cox regression analysis of DFS among cystadenocarcinoma patients

	Univariable				Multivariable			
	*p*	HR	95% CI		*p*	HR	95% CI	
Age	.031	1.065	1.011	1.135	.518	.959	.845	1.088
Gender	.168	2.539	.184	4.681				
Radical resection versus enucleation	.172	.380	.095	1.526				
Tissue invasion	.016	2.539	1.184	3.681	.039	1.286	1.035	1.554
Presence of ovarian like stroma	.042	.113	.014	.925	.196	.237	.027	2.105
Lymphatic infiltration	.045	1.781	1.474	5.279	.046	1.636	1.331	8.083

## Discussion

The current study was conducted to study clinical, laboratory, and radiological features in patients with cystadenoma compared to others with cystadenocarcinoma. Apparently, the current literature is poor with studies handling the same perspective, and most of the existing studies included a smaller sample size compared to ours. This poses an advantageous point in favor of our study.

Our findings showed a significant association between old age and malignant changes in cystic liver neoplasms. In line with our findings, Jwa and Hwang also reported a significantly older age in the malignant group compared to the benign one (median = 68 and 60 years respectively — *p* = 0.044) [2]. Arnaoutakis et al. also confirmed the previous findings [12]. Association between older age and malignancy could support the hypothesis regarding the ability of cystadenoma to turn malignant with time [5]. Lee and his associates denied the previous differences between the benign and malignant lesions regarding age [13].

In our study, males were more prevalent in the malignant group compared to females (*p* = 0.006). Another study also reported a higher prevalence of male gender in the malignant group, as they formed 28.5% of the malignant cases compared to only 4.35% of patients in the benign group (*p* = 0.048) [2]. Xu and his associates confirmed the previous findings regarding the string relation between male gender and malignant liver cystic disease (*p* = 0.01) [6].

Our findings showed that smoking was a significant risk factor for having a malignant liver cyst. Although little is known about the relationship between smoking and cystic liver neoplasms, several compounds of cigarette smoke have been shown to have a carcinogenic effect in preclinical studies, including cholangiocarcinoma [14].

We noted no significant impact of disease duration on the malignant potential (*p* = 0.2), and that was also reported by another study [6].

In our study, all malignant cases expressed abdominal pain, compared to only 62% in the benign group. Abdominal pain was strongly associated with malignancy (*p* = 0.012). Contrarily, Xu et al. reported no significant differences between the benign and malignant cases regarding their complaint (*p* = 0.16). Abdominal pain was reported by 68.29% and 70.59% of patients in the benign and malignant groups, respectively [6]. Although other authors denied any significant difference between the benign and malignant groups regarding abdominal pain, the malignant group reported a higher incidence of abdominal fullness (37% vs. 18.1% in the benign group — *p* = 0.021) [12].

In the current study, the benign and malignant groups showed comparable preoperative laboratory features. This included liver function tests and tumor markers (AFP, CEA, and CA 19-9).

Although Jwa and Hwang noted no significant differences between benign and malignant cases regarding most of the tested laboratory values, the same authors reported a significant increase in ALT and alkaline phosphatase levels in the malignant group. The former had mean values of 33.71 and 17.04 IU/L, while the latter had mean values of 103.57 and 63.09 IU/L in the malignant and benign groups, respectively [2].

Other authors also noted no significant statistical difference between the benign and malignant groups regarding laboratory values, including tumor markers. Nonetheless, the same authors reported a significant rise in both ALT and bilirubin levels in association with cystadenocarcinoma (*p* < 0.05) [6].

Sang et al. reported that most of the tested tumor markers, including CEA, AFP, CA 242, and CA 153, showed no significant difference between the benign and malignant groups. However, CA 19-9 had increased levels in both groups (838.4 μ/ml in the benign group, 337.9 μ/ml in the malignant group — *p* = 0.735) [5].

Arnaoutakis et al. reported no significant difference between the two groups regarding ALT and bilirubin levels like our study, but the authors reported a significant rise of CEA in association with malignancy (4.3 vs. 2.4 ng/ml in the benign group — *p* < 0.001) [12].

Our findings showed the presence of malignancy in smaller cysts (*p* = 0.001). Cyst size had mean values of 10 and 5 cm in the benign and malignant groups, respectively. We think that the higher prevalence of symptomatic cases in the malignant group could attribute to early seeking for the source of their symptoms, and thus, detecting the cyst in a smaller stage. Likewise, Xu and his coworkers noted a significant decrease in cyst size in the malignant group (7.1 vs. 11.7 cm in the benign group — *p* < 0.001) [6]. On the other hand, others reported no significant relation was noted between cyst size and the underlying malignancy [2], as tumor size was statistically comparable between the benign and malignant groups (*p* = 0.84).

We did not detect any significant difference between the two groups regarding the incidence of cyst septations, which is in accordance with the previous results. Other researchers also noted a comparable incidence of internal septations between the benign and malignant groups (70.73% and 76.47%, respectively — *p* = 0.58) [6].

In the current investigation, cyst loculations were present in 80% and 100% of the benign and malignant groups, respectively (*p* = 0.193). Another study confirmed our findings regarding loculations, which were present in 66.7% and 55.7% of patients in the malignant and benign groups, respectively, with no significant difference between the two groups (*p* = 0.093) [12].

In our study, the presence of mural nodules was strongly associated with malignancy (*p* = 0.033). Others reported that the presence of a mural nodule is a known risk for having cystadenocarcinoma [2, 6, 12]. Like ductal adenocarcinoma, these nodules arise from the epithelial covering and should raise the physician’s suspicion regarding an underlying malignancy [15, 16].

In the current study, the presence of solid components inside the cyst was highly suggestive of malignancy (*p* = 0.04), and this coincides with another study that denied the presence of these solid components in all benign cases [2].

We noted a significant difference between the benign and malignant cases regarding cyst calcifications (*p* = 0.018). In the same context, another study reported that cyst calcifications were detected in 14.63% and 20.59% of patients in the benign and malignant groups, respectively, with no significant difference between the two groups (*p* = 0.50) [6]. On the other hand, other studies reported that the presence of calcifications was highly suggestive of cystadenocarcinoma [9, 12, 17].

Our results showed a significant increase in the incidence of intrahepatic biliary dilatation in association with malignancy (*p* = 0.013). Another study also reported that intrahepatic biliary dilatation was encountered in 71% of the malignant cases, while the authors did not describe this finding in the malignant group [2]. Contrarily, Arnaoutakis et al. reported that the same finding was detected in 29.6% and 16.3% of patients in the malignant and benign groups, respectively, with no significant difference between the two groups (*p* = 0.913) [12].

In our study, most of the malignant lesions were in the left lobe, making it a favorable site for malignant hepatic cysts (*p* = 0.012). Although left lobe lesions were more encountered in the malignant group in the study of Jwa and Hwang (71% vs. 43% in the benign group), that difference was considered insignificant on statistical analysis (*p* = 0.39) [2]. However, other surgeons reported a higher incidence of malignant lesions in the right lobe cysts, as right lobe lesions formed 51.9% of the malignant cases compared to 28.1% in the benign group (*p* = 0.033) [12]. Furthermore, another study also reported no significant impact of lesion location on its chance of harboring malignancy (*p* = 0.24) [6].

Interestingly, we create a score for discrimination between BCA and BCAC. Score best cut off value was 2.25 for prediction of BCAC among studied patients, with 89.1% sensitivity, 100% specificity.

Regarding the operation performed in the current study, enucleation was most performed for the benign cases (76%), while the remaining cases were managed via radical resection (12%) and deroofing (12%). Radical resection was mostly performed for the malignant lesions (66.7%), whereas the remaining cases were managed by enucleation.

In fact, most surgeons recommend complete resection of these lesions if malignancy is suspected, as there is no clear preoperative test to distinguish that difference [4, 18, 19]. As partial resection is associated with high recurrence rates, one should suspect these neoplastic cystic lesions in any patient with a previous liver cyst managed by partial resection or drainage and presenting with recurrence [18, 19].

Despite the previous facts, enucleation could be performed for these cases if radical excision may risk the major nearby vascular or biliary structures, with satisfactory outcomes if the resection margins are free [6].

We noted no significant increase in intraoperative blood loss in the malignant group (*p* = 0.6). Arnaoutakis et al. did not agree with our findings regarding intraoperative blood loss, which was increased in association with malignancy (500 vs. 300 ml in the benign group — *p* = 0.026) [12].

We detected bile leakage in 6% and 8.3% of patients in the benign and malignant groups, respectively (4/62 — total incidence 6.5%). This is near the previous incidence reported by Xu et al., who reported that the same complication was noted in three cases out of the included 75 patients undergoing surgery for the same liver pathology [6].

Both of our study groups had a comparable post-operative hospital stay. Other authors confirmed the previous findings [12].

In our study, the BCA group, an increased likelihood of recurrence was associated with the type of surgical procedure performed. Specifically, the incidence of recurrence was 100% among patients who underwent an unroofing/fenestration. So complete resection of the BCA is the standard for management.

The overall incidence of recurrence in our study was generally higher among patients with BCAC (91.7%) versus BCA (12%) (*p* = 0.001), regardless of the procedure performed. Also, median disease free survival (DFS) was 36 months, whereas 1-year, 3-year, and 5-year DFS was 100%, 33.3%, and 8.3%, respectively.

Jwa and Hwang reported recurrence of the malignant condition in two out of the included seven patients with cystadenocarcinoma (28.57%). These two cases were discovered at 6- and 12-month follow-up visits [2]. Another study handling the same perspective reported that the median overall survival was 76.2 months, while 1-, 3-, and 5-year survival was 88%, 68.7%, and 45.8%, respectively [6].

We also found in the BCAC group, tissue invasion and lymphatic infiltration were independent predictors for shorter DFS. Nakajima et al. confirmed our results; they divided BCAC into two growth types according to clinicopathologic features: noninvasive (carcinoma cells confined to the cystic lesions) and invasive (carcinoma cells extending into the hepatic parenchyma or neighboring organs) [20]. In their study, patients with noninvasive tumors had a lower recurrence rate and better long-term outcome than those with invasive tumors.

In our study, after exclusion of patients misdiagnosed as simple cyst, the recurrence rate after complete resection of BCA was 0%. Simo et al. supported our conclusion that complete resection is the treatment of choice for any suspected non-invasive or invasive mucinous cystic neoplasm of the liver [21].

Our study has some limitations; the investigation was retrospective in nature, collected small sample size from a single surgical institution, mostly due to rarity of this cystic neoplasm of the liver. Also, a small group of 12 BCAC patients may carry some bias when compared with 50 BCA patients; however, we are aiming to be an addition to a later systemic review analysis to add more to this rare cystic neoplasm. Hence, more studies, including more cases from multiple hepatobiliary centers, should be conducted.

## Conclusion

Based on our previous findings, old age, male gender, smoking, the presence of symptoms, small cyst size, left lobe location, and the presence of mural nodules or solid components are suggestive for the biliary liver cyst to harbor malignancy. Also, complete surgical resection of cystic hepatobiliary tumors is necessary to obtain histopathological assessment, which is essential for the malignant potential of the lesion and for prolonged survival.

## Data Availability

The data that support the findings of this study are available from the corresponding author, upon reasonable request.
